# Evaluation of Chemcatcher® passive samplers for pesticide monitoring using high-frequency catchment scale data

**DOI:** 10.1016/j.jenvman.2022.116292

**Published:** 2022-12-15

**Authors:** Luke G. Farrow, Phoebe A. Morton, Rachel Cassidy, Stewart Floyd, W. Colin McRoberts, Donnacha G. Doody, Philip Jordan

**Affiliations:** a Agri-Environment Branch, Agri-Food and Bioscience Institute, Belfast, UK; b Food Research Branch, Agri-Food and Bioscience Institute, Belfast, UK; c School of Geography and Environmental Sciences, Ulster University, Coleraine, UK

**Keywords:** Chemcatchers®, Passive sampling, High frequency sampling, Acid herbicides, Water quality monitoring, Catchment-scale

## Abstract

Passive samplers (PS) have been proposed as an enhanced water quality monitoring solution in rivers, but their performance against high-frequency data over the longer term has not been widely explored. This study compared the performance of Chemcatcher® passive sampling (PS) devices with high-frequency sampling (HFS: 7-hourly to daily) in two dynamic rivers over 16 months. The evaluation was based on the acid herbicides MCPA (2-methyl-4-chlorophenoxyacetic acid), mecoprop-P, fluroxypyr and triclopyr. The impact of river discharge parameters on Chemcatcher® device performance was also explored. Mixed effects modelling showed that time-weighted mean concentration (TWMC) and flow-weighted mean concentration (FWMC) values obtained by the HFS approach were both significantly higher (p < 0.001) than TWMC values determined from PS regardless of river or pesticide. Modelling also showed that TWMC_PS_ values were more similar to TWMC_HFS_ than FWMC_HFS_ values. However, further testing revealed that MCPA TWMC values from HFS and PS were not significantly different (p > 0.05). There was little indication that river flow parameters altered PS performance—some minor effects were not significant or consistent. Despite this, the PS recovery of very low concentrations indicated that Chemcatcher® devices may be used to evaluate the presence/absence and magnitude of acid herbicides in hydrologically dynamic rivers in synoptic type surveys where space and time coverage is required. However, a period of calibration of the devices in each river would be necessary if they were intended to provide a quantitative review of pesticide concentration as compared with HFS approaches.

## Highlights

• Chemcatchers® were evaluated for monitoring acid herbicides in rivers.
• Presence/absence and magnitude were compared with high frequency data.
• High frequency concentrations were mostly always higher than Chemcatcher®.
• Chemcatcher® performance was not significantly influenced by river flow parameters.
• The higher concentrations of MCPA were best captured by Chemcatchers®.

## Graphical abstract

**Figure:**
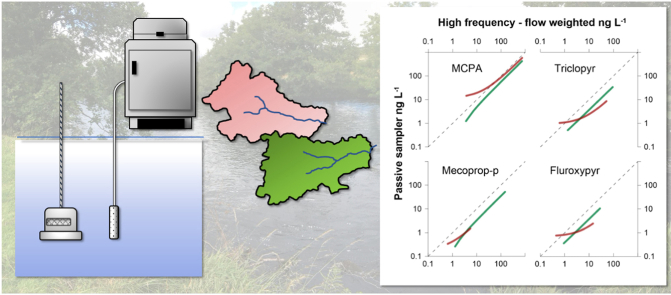

## 1 Introduction

The Driver-Pressure-State-Impact-Response model is used as an environmental policy review framework where monitoring is a key component (Ness et al., 2010). Emphasis is placed on ‘state’ variables in environmental systems, and a common optimisation is to trade-off monitoring sites at a high temporal resolution and instead implement a high spatial resolution regime (Strobl and Robillard, 2008). This is particularly robust when spatial correlation between sites is high and temporal correlation is low (Rhodes and Jonzén, 2011). In river systems under regulatory requirements, this ‘state’ monitoring translates to low frequency sampling at many sites where longer-term trends can be assessed for both physico-chemical and biological variables (Dupas et al., 2019; Hering et al., 2010; Saad et al., 2011).

However, two drawbacks of this approach have been identified in the literature, particularly in relation to physico-chemical variables. First is the inability of low resolution sampling to resolve complex relationships between dynamic river flows and variable concentrations —relationships that can aid in the development of water quality models (Haygarth et al., 2004). This can also provide understanding of pollution sources through concentration-discharge (C-Q) relationships (Sherriff et al., 2016). Second, ‘state’ type monitoring may not be suitable in many river water quality scenarios to capture the immediate ‘impact’ of mitigation policies that are designed for high magnitude, short duration (i.e., acute) pollution processes (Westerhoff et al., 2022). While high-frequency water quality monitoring approaches can help with both issues (Rode et al., 2016), the capital and data management expense of a high spatial coverage can be prohibitive beyond focused research studies (but see Jones et al. (2018) for an example of state-wide nitrate sensor investment in the USA).

Passive samplers, such as the Polar Organic Chemical Integrative Sampler (POCIS) (Bernard et al., 2019; Van Metre et al., 2017) or Chemcatcher® devices (Moschet et al., 2015; Novic et al., 2017; Taylor et al., 2020) potentially offer an optimum solution between high-spatial coverage but low temporal resolution, and low spatial resolution but higher temporal resolution options. Such devices, which are now commercially available, are deployed to determine the time-average or time-weighted mean concentration (TWMC) of a variety of contaminants of interest in waterbodies by adsorption to media in the field, and elution in the laboratory. The TWMC differs from the flow-weighted mean concentration (FWMC) of a variable as the latter weights the concentration by both the time-period it represents and the flow occurring during that time. This is more representative of the dynamics of a surface-water driven river system where there is a high flow dependency of concentrations.

However, validation of both TWMC and FWMC with passive samplers is rarely reported, or is constrained due to combinations of the cost of analytical processes for some variables, such as pesticides, and the catchment scale of study. For example, in a detailed analysis of Chemcatcher® device performance on capturing TWMCs of acid herbicide in the UK by Townsend et al. (2018), field trials were only over a short time period (14–17 days) and compared against seven samples at sites within the 1530 km^2^ River Exe catchment. Additionally, an analysis of SorbiSystems™ SorbiCell passive samplers, which offer flow proportionality during the period of deployment through the dissolution rate of a tracer in addition to the adsorption of the contaminant of interest (Vendelboe et al., 2012), showed a limited relationship to phosphorus (P) and nitrogen (N) FWMC measured by high resolution monitoring in small (5 km^2^) flashy, river catchments; although TWMC comparisons were better (Jordan et al., 2013). Rozemeijer et al. (2016) reported SorbiCell data as TWMC for P and N in 1 ha plot trials over several years, but these were always over-predictions.

Both TWMC and FWMC assessments of passive samplers are important for water utilities as validation of passive samplers enables them to use these to increase both spatial and temporal sampling resolution in source water catchments. For example, TWMC provides a complimentary measurement of abstracted water state from grab samples where the abstraction rate is constant, or at least not in proportion to the river flow rate. In catchments with many tributaries, TWMCs by passive samplers can provide important synoptic insights for risk assessments and spatial targeting of mitigation measures in the same way that those based on grab-sampling do (Morton et al., 2021). However, FWMCs provide equally important catchment insights into pollution flux dynamics over the longer term and particularly where those pollutants have a flow dependency for mobilisation (Cassidy et al., 2018; Ouyang, 2021). Assessments of each should be equally valid for ‘state’ and ‘impact’ monitoring requirements.

Therefore, in large catchments, a clear knowledge gap is for longer term testing of passive samplers against both the TWMC and FWMCs of analytes of concern that are prohibitively expensive (or technologically difficult) to measure continuously at high resolution. In this study, these issues were addressed in Irish rivers with the aim of assessing the performance of off-the-shelf Chemcatcher® passive samplers, which are designed to give TWMCs, for acid herbicide monitoring. Elevated concentrations of four acid herbicides (MCPA (2-methyl-4-chlorophenoxyacetic acid), triclopyr, fluroxypyr and mecoprop-P) have been recorded in surface waters throughout Ireland (Zhao et al., 2013; Khan et al., 2020; Morton et al., 2021), and are a burden on water treatment to meet drinking water standards. In 2017, in Northern Ireland, these were the four most commonly used (by mass) herbicides and together they accounted for 83% of the total mass of the 40 most used herbicides applied (Lavery et al., 2017). In the Republic of Ireland, they were amongst the 10 most used (by mass) herbicides, accounting for 41% of the total mass of the 40 most used herbicides (Pesticide Control Division, 2017).

The objectives of the study were to 1) develop paired high-resolution herbicide concentration datasets synchronous with river discharge and passive sampler deployments and, 2) assess the TWMC and FWMC data collations against passive sampler results.

## 2 Methods

### 2.1 Study area

**Fig. 1:**
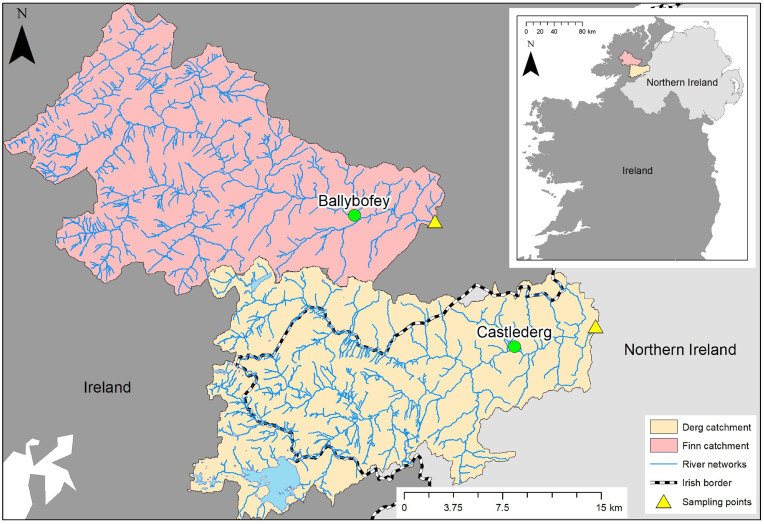
Map showing the locations of the two study catchments and the monitoring stations. The inset highlights the location of the catchments on the island of Ireland

The study was conducted in the River Derg and River Finn, which are large, surface-water driven catchments in the cross-border area of north-west Ireland (Fig. 1). The River Derg is used as a drinking water source with the abstracted water treated and distributed to a population of approximately 40,000 people. Both rivers are important wild salmon and trout fisheries. Catchment characteristics are shown in Table 1.

**Table 1:** Characteristics of the two river catchments used for passive sampler assessment in the border area of north-west Ireland.

		River Derg	River Finn
Catchment area to monitoring outlet, km^2^		384	386
Grid reference		54.722ᵒ N, 7.497 ᵒ W	54.795ᵒ N, 7.686 ᵒ W
Lithology (channel to uplands)		Luvisol, Cambisol, Umbrisol, blanket peatland	Luvisol, Cambisol, Umbrisol, Gleysol, blanket peatland
Geology		Metasediments/granites	Metasediments/schists/quartzite/granites
Land use, %^a^			
Agriculture		35.4	25.8
Forest		17.6	11.2
Peatland		44.0	61.6
Other		3.0	1.4
River flow (2017–2019 data)^b^			
Annual river flow, mm		1890	1520
Min flow, m^3^ s^−1^		1.02	1.94
Max flow, m^3^ s^−1^		203	210
Median flow, m^3^ s^−1^		11.1	12.9
Q5:Q95 ratio^c^		39.4	24.9

a Copernicus (2019).

b River Derg data from the National River Flow Archive, Centre for Ecology and Hydrology, UK. River Finn data from the Office of Public Works, Ireland.

c The Q5 value (95th percentile of flow) is the flow volume that was matched or exceeded in 5% of observations during the period of observation. The Q95 (5th percentile of flow) is the flow volume that was matched or exceeded in 95% of observations during the period of observation.

### 2.2 Sample collection

Both high-frequency water quality monitoring equipment and Chemcatcher® devices were deployed at the catchment outlets (Fig. 1) between October 30, 2018 and February 25, 2020 (i.e., almost 16 months). For high-frequency sampling (HFS), refrigerated samplers (6712FR, Teledyne ISCO, USA) were used to collect samples every 7 h during the spring, summer and autumn and daily in winter (See Table S1 for dates). 7-hourly sampling was in accordance with the “Plynlimon” approach (Halliday et al., 2012), as evaluated by Jordan and Cassidy (2011) whilst the winter sampling frequency was reduced to daily as pesticide concentrations were expected to be lower at this time of year.

All samples were refrigerated within 8 h of collection from the autosampler and were analysed within 3 days of receipt. Samples were extracted and concentrated (Gervais et al., 2008) then analysed by LC-MS/MS (see Morton et al. (2021) for details of storage and performance tests). See Supplementary materials Table S2 for analytical limits of detection.

For passive samplers (PS), a deployment period of 14 days was adopted, following the field study of Townsend et al. (2018). Deployments occurred between October 30, 2018 and February 20, 2020 (See Supplementary materials Table S3 for details). All PS were supplied and analysed by Natural Resources Wales (NRW; Swansea University, UK) under a commercial contract. NRW labs are accredited according to ISO/IEC 17025:2017 by the UKAS. Until the July 9, 2019, Empore™ design Chemcatcher® bodies (A T Engineering, Tadley, UK), including an Empore™ anion-SR disk acting as the receiving phase (3M™, Bracknell, UK) and a Polyethersulfone (PES) (Supor® 200, 0.2 μm pore diameter disks) (Pall Europe Ltd, Portsmouth, UK) diffusion membrane were used. For all subsequent deployments (July 23, 2019 to February 25, 2020), the supplier of the anion-SR disk was changed to CDS Analytical (Oxford, USA) whilst all other parts remained the same. The impact of the change in anion-SR disk was evaluated by NRW and, where necessary, changes were made to the calculations used to determine PS TWMC, ensuring comparability of results obtained before and after the change (J. Pearce, Pers. Comm.). The LODs for both variants of chemcatcher® disk are detailed in Table S2.

PS deployments were undertaken in accordance with the standard operating instructions provided by NRW to end users. Specifically each fortnightly deployment required the placement of three devices, housed within a steel cage, into the river. A fourth device was also exposed to the air to account for airborne pesticide contaminants and to act as a control. Cages were securely attached to a fixed point on the river bank such that they were at least 50 cm underwater but at least 30 cm off the river bed in order to ensure that they were constantly within the main river flow. It was not possible to analyse the first batch of PS devices deployed in the Finn as a series of extreme high flows disassembled the devices and destroyed the SR disks (see subsequent statistical method development in section 2.4.).

River discharge measurements were taken from gauging stations located approximately 9 km upstream of the HFS/PS monitoring points (Derg: Department for Infrastructure (2020); Finn: Office of Public Works (2020)) following Atcheson et al. (2022).

### 2.3 Data handling

Analyses were performed in R (R Core Team, 2020). Values below the LOD were set as half the LOD in the analyses (USEPA and Information, 2000).

For each deployment, fortnightly TWMCs were calculated for both PS and HFS monitoring techniques. FWMCs were also calculated for the same periods from the HFS dataset.

The PS time-weighted mean concentration (TWMC_PS_) was calculated as:

[1] $${TWMC}_{PS}=\frac{M_{s}}{R_{s}t_{i}}$$

where *M*_*s*_ is the mass (μg) of analyte in the sampler*, R*_*s*_ is the sampler uptake rate (L day^−1^), and *t*_*i*_ is the exposure time (days).

The HFS time-weighted mean concentration (TWMC_HFS_) was calculated as:

[2] $${TWMC}_{HFS}=\frac{\sum_{1}^{n}\left(c_{i}*t_{i}\right)}{\sum_{1}^{n}t_{i}}$$

where *c*_*i*_ is the pesticide concentration in the *i*th sample (μg L^−1^) and *t*_i_ is the sampling interval (days).

The HFS flow-weighted mean concentration (FWMC_HFS_) was calculated as:

[3] $${FWMC}_{HFS}=\frac{\sum_{1}^{n}\left(c_{i}t_{i}q_{i}\right)}{\sum_{1}^{n}\left(t_{i}q_{i}\right)}$$

where *q*_*i*_ is the flow in the *i*th sample period (m^3^).

For each PS deployment, the fortnightly pesticide load, L_PS_ (kg), was calculated following Rozemeijer et al. (2010) and Novic et al. (2017) as:

[4] $$L_{PS}={TWMC}_{PS}*Q_{i}*t_{i}$$

where *Q*_*i*_ is the average flow during the deployment period *i* (m^3^ s^−1^) and *t*_*i*_ is the sampling interval (s).

The HFS pesticide loads (L_HFS_) during each corresponding fortnight, were calculated by linear interpolation of the 7-h time series:

[5] $$L_{HFS}=\sum_{1}^{n}\left(c_{i}t_{i}q_{i}\right)$$

Linear interpolation was considered appropriate as there is generally serial correlation between successive measurements due to the dominance of diffuse inputs, and this has been validated for other diffuse pollutants in similarly dynamic river systems (Jordan and Cassidy, 2011).

### 2.4 Statistical analysis

Data were analysed using mixed effects models and multiple regressions in R (R Core Team, 2020). The analysis is briefly described here; for full details, see the Statistical Analysis section in the supporting information. Differences between TWMC_PS_, TWMC_HFS_ and FWMC_HFS_ were analysed on log-transformed data using linear mixed effects models to test for effects of the sampling and calculation methods. The fixed effects in the model were Sites, Pesticides, PS Disk-type and the interactions between Method and Site and Method and Pesticide. The end date of the fortnightly period was included as a random intercept and Pesticide as a random slope. A variance structure using Pesticide was also included. A second separate analysis was developed for loads (comparing L_PS_ and L_HFS_) using the same fixed and random effects (but without disk-type). These analyses were run using a backward stepwise selection approach with the significance of the final model structure being compared against α = 0.05 (“lmer” and “lmerTest” packages (Kuznetsova et al., 2017). A post-hoc test was applied to determine significant differences between the Methods and between factors in the Method and Pesticide interaction term.

Additionally, percentage differences between TWMC_PS_ and TWMC_HFS_ between TWMC_PS_ and FWMC_HFS_ were assessed against changing river discharge metrics (Q_min_, Q_max_, Q_median_, and Q_mean_) on data from both sites separately and combined. Variance inflation factors (VIFs) were used to assess collinearity with stepwise removal of variables until all VIFs were below 1/(1-R^2^ of model) or until only two variables were left (single regressions were also checked).

## 3 Results

### 3.1 Patterns in pesticide concentrations and PS performance

**Fig. 2:**
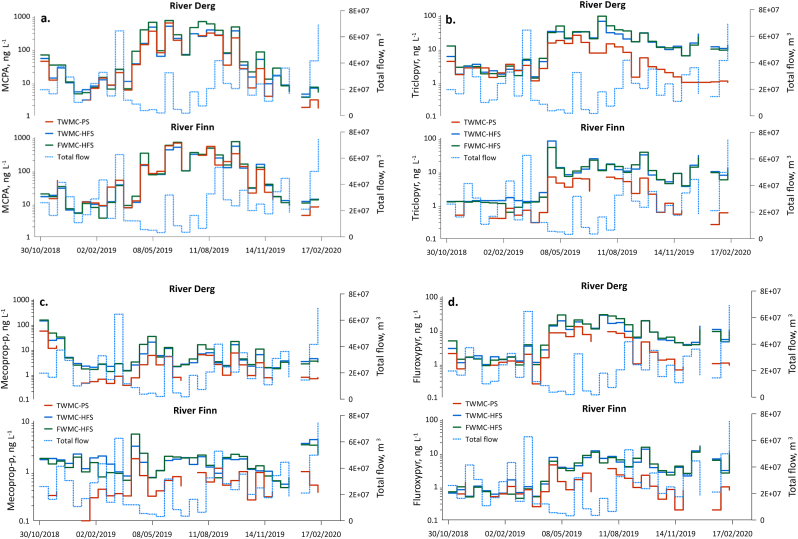
Fig. 2 a-d Comparison of PS and HFS TWMC and FWMC values for each of the four pesticides in each catchment through time. All HFS samples for Triclopyr in the Finn catchment taken before 2nd April 2019 were below the limit of detection

**Fig. 3:**
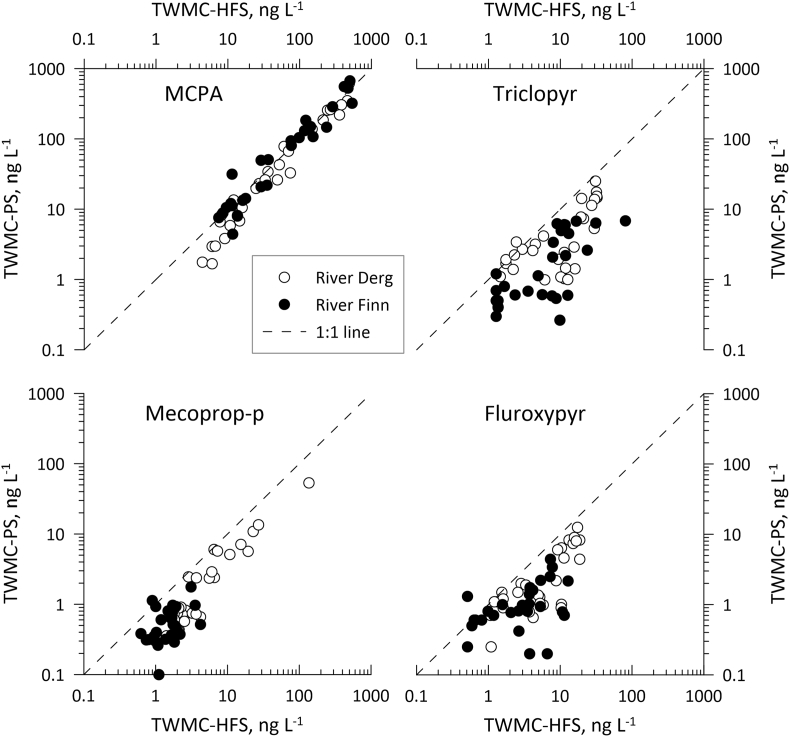
Relationship between TWMC_HFS_ and TWMC_PS_ values for each pesticide in relation to the 1:1 line. All HFS samples for Triclopyr in the Finn catchment taken before 2nd April 2019 were below the limit of detection

**Fig. 4:**
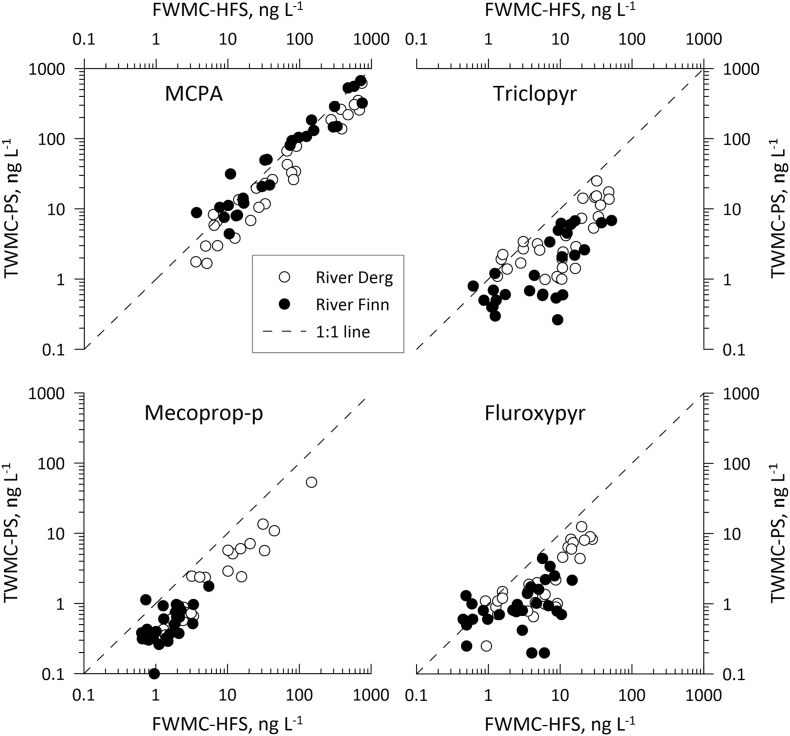
Relationship between FWMC_HFS_ and TWMC_PS_ for each pesticide in relation to the 1:1 line. All HFS samples for Triclopyr in the Finn catchment taken before 2nd April 2019 were below the limit of detection

**Fig. 5:**
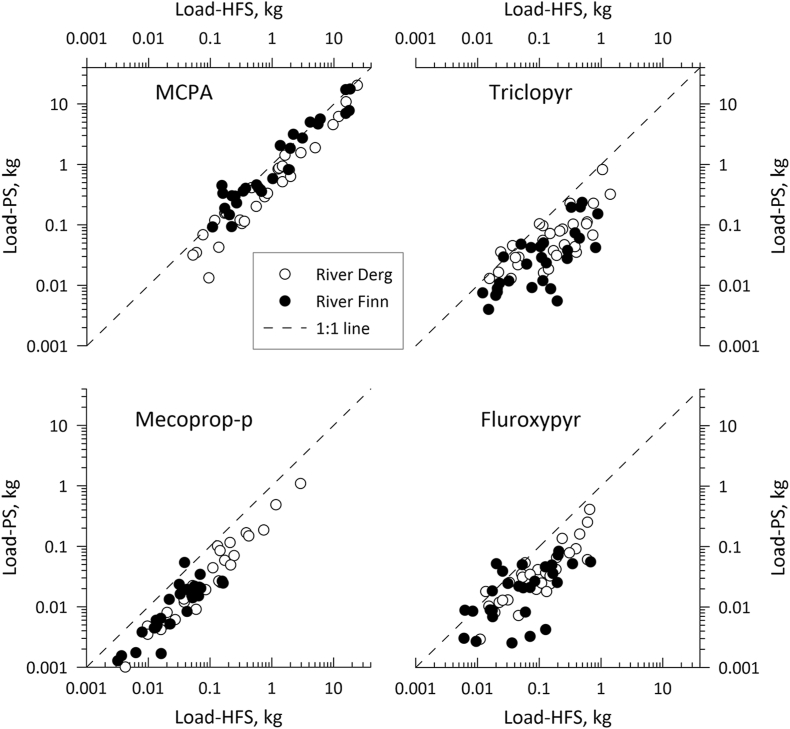
Pesticide loads in each monitoring period, as determined by HFS and PS techniques, relative to the 1:1 line. All HFS samples for Triclopyr in the Finn catchment taken before 2nd April 2019 were below the limit of detection

MCPA TWMCs and FWMC_HFS_ values were higher in both rivers than those for any other pesticide (Table S4). In the Derg, fortnightly TWMC values varied between 1.68 and 619 ng L^−1^ (TWMC_PS_) and 4.43 and 493 ng L^−1^ (TWMC_HFS_) and FWMC_HFS_ concentrations ranged between 3.56 and 734 ng L^−1^. In the Finn, TWMC values similarly varied between 4.43 and 668 ng L^−1^ (TWMC_PS_) and 5.32 and 537 ng L^−1^ (TWMC_HFS_), and FWMC_HFS_ varied between 3.67 and 744 ng L^−1^ (Fig. 2a, Table S4). MCPA was the only pesticide for which peak spray period TWMCs (HFS and PS) and FWMC_HFS_ were in excess of 100 ng L^−1^ (the legal maximum permitted concentration of a single pesticide in treated drinking water in the EU (Council of the European Commission, 1998)). Fortnightly MCPA loads in the Derg varied between 0.04 and 24.0 kg (L_HFS_) and 0.01 and 20.3 kg (L_PS_) and varied between 0.06 and 18.2 kg (L_HFS_) and 0.09 and 17.6 kg (L_PS_) in the Finn (Table S4). Comparison of TWMC_PS_ and L_PS_ values with TWMC_HFS_, FWMC_HFS_ and L_HFS_ values for the same periods showed positive relationships for all in both catchments (Fig. 3, Fig. 4, Fig. 5). MCPA PS values in the Finn exceeded the TWMC_HFS_ values on 16 occasions, particularly between January and July 2019 when all TWMC_PS_ values were more than TWMC_HFS_ values (Table S4). There were also three occasions when the TWMC_PS_ value was more than 1.5 times that of the TWMC_HFS_ value. TWMC_PS_ values were also greater than FWMC_HFS_ on 11 occasions, again predominantly between January and July 2019. In the Derg TWMC_PS_ values were greater than TWMC_HFS_ on five occasions between early February and early August 2019 and FWMC_HFS_ on two occasions (late February and early July 2019). TWMC_PS_ values were never more than 1.5 times that of the equivalent HFS values and neither catchment recorded a PS value that was less than half of the equivalent HFS value.

Triclopyr TWMC_HFS_ values ranged between 1.46 and 65.3 ng L^−1^, FWMC_HFS_ values were between 1.33 and 93.0 ng L^−1^ and TWMC_PS_ were between 0.99 and 25.1 ng L^−1^ in the Derg, and were between 1.28 and 80.8 ng L^−1^ for TWMC_HFS_, 0.61 and 51.8 ng L^−1^ for FWMC_HFS_ and 0.26 and 6.80 ng L^−1^ for TWMC_PS_ in the Finn (Fig. 2b, Table S5). Triclopyr loads in the Derg varied between 0.02 and 1.68 kg (L_HFS_) and 0.01 and 0.82 kg (L_PS_), and varied between 0.01 and 0.88 kg (L_HFS_) and <0.01 and 0.24 kg (L_PS_) in the Finn (Table S5). Comparison of TWMC_PS_ and L_PS_ values with TWMC_HFS_, FWMC_HFS_ and L_HFS_ values for the same periods showed positive relationships for all in both catchments (Fig. 3, Fig. 4, Fig. 5). The TWMC_PS_ value was larger than TWMC_HFS_ values in the Derg on two consecutive periods between late January and mid-February 2019, but there were no occasions when this happened in the Finn Table S5). TWMC_PS_ and L_PS_ values in the Derg were also more than FWMC_HFS_ and L_HFS_ values on three consecutive occasions between late January and early March 2019), but TWMC_PS_ and L_PS_ value were only more than FWMC_HFS_ and L_HFS_ values on one occasion in the Finn (mid-February 2019). No PS values was less than half of the equivalent HFS value throughout the study.

Mecoprop-P concentrations were the lowest of the evaluated herbicides in the Finn, ranging between 0.54 and 4.23 ng L^−1^ (TWMC_HFS_), 0.46 and 5.46 ng L^−1^ (FWMC_HFS_) and 0.10 and 1.76 ng L^−1^ (TWMC_PS_) (Table S6). In the Derg, mecoprop-P concentrations ranged between 1.42 and 136 ng L^−1^ (TWMC_HFS_), 1.28 and 148 ng L^−1^ (FWMC_HFS_) and 0.38 and 53.6 ng L^−1^ (TWMC_PS_) (Table S6). Although the highest mecoprop-P concentrations were observed at the start of the study, these rapidly decreased to close to those observed in the Finn (Fig. 2c, Table S6). Mecoprop-P loads in the Derg varied between <0.01 and 2.92 kg (L_HFS_) and <0.01 and 1.09 kg (L_PS_), and varied between <0.01 and 0.16 kg (L_HFS_) and <0.01 and 0.05 kg (L_PS_) in the Finn (Table S6). The PS values were only larger than the equivalent HFS parameter on one occasion (Finn: late August 2019) and PS values were never less than half the equivalent HFS value (Table S6). There were positive relationships between all PS and HFS parameters in both catchments (Fig. 3, Fig. 4, Fig. 5).

Fluroxypyr concentrations ranged between 0.90 and 26.8 ng L^−1^ (TWMC_HFS_), 0.88 and 28.7 ng L^−1^ (FWMC_HFS_) and 0.25 and 12.5 ng L^−1^ (TWMC_PS_) in the Derg and between 0.44 and 14.6 ng L^−1^ (TWMC_HFS_), 0.51 and 16.0 ng L^−1^ (FWMC_HFS_) and 0.20 and 4.40 ng L^−1^ (TWMC_PS_) in the Finn (Fig. 2d, Table S7). Fortnightly fluroxypyr loads in the Derg varied between <0.01 and 0.65 kg (L_HFS_) and <0.01 and 0.41 kg (L_PS_), and varied between <0.01 and 0.68 kg (L_HFS_) and <0.01 and 0.08 kg (L_PS_) in the Finn (Table S7). There was a positive relationship between all PS parameters and their HFS equivalent (Fig. 3, Fig. 4, Fig. 5). TWMC_PS_ and L_PS_ values were greater than their equivalent HFS parameter in the Derg on one occasion (late February 2019) whilst in the Finn TWMC_PS_ values exceed FWMC_HFS_ values on four occasions (early December 2018, mid-January 2019 and February 2019 (two consecutive deployments)). The L_PS_ values in the Finn exceeded the L_HFS_ value in early December 2018 and from mid-January until early March 2019. The largest PS over-estimate in the Finn was more than double the HFS value whilst no PS value was less than half the HFS value. PS over-estimates in the Derg were never more than one third higher than the HFS parameter and never less than half.

### 3.2 Statistical analysis of concentration and flow variables

The AIC values for the mixed effects models indicated that the end date of the fortnightly period should be kept as a random intercept and Pesticide as both a random slope and a variance structure for both TWMC/FWMC and load models. For the fixed effects, exclusion of the interaction between Method and Site improved both models, and exclusion of the Pesticide-Disk interaction improved the TWMC/FWMC model but terms for Method, Pesticide and Site and the interaction between Method and Pesticide were retained for both. Whilst the term for Disk was retained in the final TWMC/FWMC model, it was not significant (F_1_, _26_ = 2.59, p = 0.12).

Therefore, the final models showed that TWMC/FWMCs and loads were significantly different between the Methods overall (i.e., regardless of Pesticide or Site; F_2_, _584_ = 172.6, p < 0.0001 and F_1_, _360_ = 220.1, p < 0.0001, respectively), with HFS recording higher concentrations and loads than PS. This was regardless of whether concentrations were calculated as TWMCs or FWMCs (p < 0.0001) compared to PS but not when compared to each other (p = 0.97). TWMC_PS_ were more similar to TWMC_HFS_ than FWMC_HFS_. TWMC/FWMCs and loads also differed significantly between the four pesticides overall (F_3_, _36_ = 80.5, p < 0.0001 and F_3_, _35_ = 88.8, p < 0.0001, respectively), with MCPA having the highest concentrations and loads, and between the two sites overall (F_1_, _585_ = 176.0, p < 0.0001 and F_1_, _361_ = 54.0, p < 0.0001, respectively), with Derg values being higher than Finn values. Whilst the Method-Pesticide interaction was significant for both the TWMC/FWMC and load models (F_6_, _584_ = 9.00, p < 0.0001 and F_3_, _360_ = 7.55, p < 0.0001, respectively), the post-hoc test demonstrated that the concentrations and loads derived from HFS were significantly higher than those from PS for mecoprop-P, fluroxypyr and triclopyr (p < 0.001 for all) but not for MCPA (p > 0.05) (see Fig. 4, Fig. 5).

Of all the multiple linear regressions performed, most were not significant (low R^2^, p > 0.05), meaning that very little of the difference between the sampling methods was explained by the river flow variables. Regressions for fluroxypyr initially suggested relationships between the difference in sampling methods (comparing TWMC_PS_ to both TWMC_HFS_ and FWMC_HFS_ separately) and Q_max_ for the Finn only but these were shown to be strongly influenced by one very high value and there were no relationships (p > 0.36) once this was removed. There was a significant negative relationship between Q_min_ and the percentage difference in Derg MCPA concentrations between TWMC_PS_ and TWMC_HFS_ (R^2^ = 0.17, p = 0.028), which was also shown in the multiple regression when Q_max_ was included (R^2^ = 0.23, p = 0.033), suggesting that more extreme low flows are likely to improve the Chemcatcher performance in relation to MCPA adsorption. However, there were no relationships with flow metrics for the Finn or the difference between TWMC_PS_ and FWMC_HFS_. Conversely, TWMC_PS_ and FWMC_HFS_ percentage differences in mecoprop-P concentrations for both sites combined had a positive relationship with Q_max_ in single (R^2^ = 0.10, p = 0.020) and multiple (with Q_min_; model R^2^ = 0.16, p = 0.010) regressions, with higher Q_max_ corresponding to better agreement between the two sampling methods. However, these relationships were not significant for either site separately nor for differences between TWMC_PS_ and TWMC_HFS_. Multiple regressions with the percentage difference in Finn fluroxypyr concentrations between TWMC_PS_ and both TWMC_PS_ and FWMC_HFS_ as the dependent variables suggested that there was a negative relationship with Q_min_ but only when combined with Q_median_ for differences between TWMC_PS_ and TWMC_HFS_ (R^2^ = 0.24, p = 0.030) and with Q_max_ for differences between TWMC_PS_ and FWMC_HFS_ (R^2^ = 0.28, p = 0.017). Overall, whilst there may be a small effect of flow metrics on the performance of the PS compared to the HFS samples, the effects appear minor, with low explained variability, and were not consistent.

## 4 Discussion

This study has demonstrated that, whilst the commercially available Chemcatchers® used in this study compared favourably to HFS for MCPA, the TWMCs and loads derived from Chemcatchers® for mecoprop-P, triclopyr and fluroxypyr were significantly lower, thus bringing into question their utility to completely replace HFS to quantify pesticide concentrations in highly dynamic rivers similar to those monitored here.

However, the majority of TWMC_PS_ values were within a factor of two of the TWMC_HFS_ values, which is in agreement with studies in other rivers (Moschet et al., 2015; Townsend et al., 2018) and lab studies (Schreiner et al., 2020; Shaw and Mueller, 2009). Additionally, the general linearity of the relationship between TWMC_PS_ and TWMC_HFS_ values, albeit with only MCPA concentrations being on the 1:1 Line (Fig. 3), indicates that Chemcatcher® devices may be useful in identifying general trends in pesticide concentrations and would be suitable for the determination of the presence/absence of acid herbicides in rivers. Where these devices are intended to be deployed in replacement of an HFS monitoring strategy, in-river calibration may be required (Moschet et al., 2015; Novic et al., 2017; Vermeirssen et al., 2009), or deployment alongside passive flow monitors (Fauvelle et al., 2017).

TWMCs derived from Chemcatcher® devices have not previously been compared with FWMCs from a high-frequency monitoring system, which are viewed as a more accurate representation of contaminant concentration in a waterbody, particularly in surface flow dominated river systems (Cassidy et al., 2018). The results obtained here indicate that TWMCs determined by Chemcatcher® devices are similar to FWMC_HFS_ values, but are closer to TWMC_HFS_. This is probably because passive samplers are deployed for relatively long periods of time and so will experience a variety of flow conditions during each deployment, but only uptake compounds proportionally to the concentrations (Vrana et al., 2005). Whilst the concentrations of the pesticides sampled in this study are likely to be related to flow given their solubility in water, amounts and timing of application will also play a role.

Additionally, the brevity of peaks in pesticide concentrations may also influence PS performance (Moschet et al., 2015; Shaw and Mueller, 2009) because it takes some time for a change in pesticide concentration in water to be reflected in the PS, which is referred to as the lag phase. The longer the lag phase, the greater the disparity between recorded and actual time-weighted pesticide concentration, although the lag phase is suggested to be brief for the pesticides studied here (Townsend et al., 2018). The size of the peak, relative to background pesticide concentrations has also been shown to increase the divergence between TWMC_HFS_ and TWMC_PS_ values because the lag phase reduces the opportunity for the receiving phase material to experience the highest concentrations (Shaw and Mueller, 2009).

The impact of flow velocity and biofouling on the performance of Chemcatcher® devices has previously been explored (Lissalde et al., 2016; Moschet et al., 2015). Although not explicitly tested for here, the independent flow variables used in the multiple-regression analysis could not significantly explain differences between PS and HFS and therefore biofouling may have impacted PS performance (Lissalde et al., 2016; Moschet et al., 2015). It was also observed during PS retrieval that there was often discolouration of the outer membrane, which may indicate biofouling. Harman et al. (2012) argued that there will be some inter-compound variation in PS performance because of the affinity of the individual chemicals for the biofouling agent. Where the biofouling agent accumulates the pesticides of interest more readily than the PS receiving phase, there may be an increase in the TWMC recorded compared to an unfouled PS. Field observations in this study suggested that samplers from the Derg experienced more visible contamination than those deployed in the Finn. However, greater deviation between methods was recorded in the TWMCs from the Finn suggesting this is unlikely to play a large role.

A final note of caution on passive sampler performance is also required in these interpretations. It is recognised that the adoption of the commercially available Chemcatcher® calibration curves may have also influenced the TWMC values obtained, compared with results obtained from a locally-derived calibration curve. However, the face-value approach used in this study is likely to be representative of deployments by the majority of users obtaining Chemcatchers® via a commercial route. Nevertheless, programmes that use Chemcatcher® devices, should also consider whether the costs of undertaking a local calibration exercise (including recovery estimates) are justified for the purpose of the study.

## 5 Conclusions

Passive samplers are an important enhanced monitoring method where information beyond water quality ‘state’ may be required in a Driver-Pressure-State-Impact-Response framework. For synoptic surveys, for example where many tributaries of a river system require a presence/absence/magnitude comparison for Driver-Pressure-State-Impact-Response ‘impact’ monitoring, passive samplers are likely to be an optimum solution. In this regard, Chemcatcher® devices have been shown in this study to reliably document the presence/absence of specific pesticides at very low concentrations. However, the PS values (for both TWMC and Load) in this paired catchment study were significantly different from the HFS values for three of the four pesticides (with no further explanation from exposure to different river flow conditions) indicating that Chemcatcher® devices cannot replace an HFS approach without further, local calibration. A possible exception is for MCPA, or for other pesticides at higher concentrations, although further work is required to explore this further.

## Author statement

**Luke Farrow**: Investigation, Formal analysis, Writing – original draft, Writing – review & editing, Visualisation. **Phoebe Morton**: Investigation, Methodology, Formal analysis, Writing – review & editing, Visualisation. **Rachel Cassidy**: Conceptualisation, Methodology, Formal analysis, Writing – review & editing, Visualisation, Funding acquisition. **Stewart Floyd**: Methodology, Validation, Writing – review & editing. **W. Colin McRoberts**: Conceptualisation, Writing – review & editing, Supervision, Funding acquisition. **Donnacha G. Doody**: Conceptualisation, Writing – review & editing, Supervision, Funding acquisition. **Philip Jordan**: Conceptualisation, Methodology, Investigation, Writing – review & editing, Supervision, Funding acquisition.

## Declaration of competing interest

The authors declare that they have no known competing financial interests or personal relationships that could have appeared to influence the work reported in this paper.

## Data Availability

Research data is included in the supplementary section.
