# Supplementary material for: Evaluation of Chemcatcher® passive samplers for pesticide monitoring using high-frequency catchment scale data
Source: J Environ Manage. 2022 Dec 15;324:116292. doi: 10.1016/j.jenvman.2022.116292 (PMC9666346; doi:10.1016/j.jenvman.2022.116292)
Supplement: Multimedia component 1 [file mmc1.pdf]

Statistical analysis

Differences between $TWMC_{PS}$, $TWMC_{HFS}$ and $FWMC_{HFS}$ were analysed on log-transformed data using linear mixed effects models to test for effects of the sampling and calculation methods. Sites (Derg or Finn) and Pesticides (MCPA, mecoprop-P, fluroxypyr or triclopyr) and the interactions between Method and Site and Method and Pesticide were also included as fixed effects, as was passive sampler disk-type (Empore™ or CDS). The baseline regression model was fitted using the "gls" function from the "nlme" package in R (R Development Core Team 2020).

Random effects were included by refitting the models using the "lme" function in the "nlme" package. The end date of the fortnightly period was included as a random intercept and Pesticide as a random slope. A variance structure using Pesticide was also included. A second separate analysis was developed for loads (comparing L_{PS} and L_{HFS}) using the same fixed and random effects (but without disk-type).

These analyses were run using a backward stepwise selection approach and the lowest Akaike Information Criterion (AIC) value used to determine the model with the better fit (Zuur et al. 2009). Random effects were tested against one another using a restricted maximum likelihood function before fixed effects were evaluated with maximum likelihood functions. The significance of the final model structure was compared against $\alpha = 0.05$ ("lmer" and "lmerTest" packages (Kuznetsova et al. 2017), including the Satterthwaite correction for an unbalanced design (Spilke et al. 2005) due to the missing Finn PS results). The "glht" function with the "Tukey" option from the "multcomp" package (Hothorn et al. 2008) was used as a post-hoc

test to determine significant differences between the Methods and between factors in the Method and Pesticide interaction term.

Additionally, as time was a fixed variable during PS deployments (14 days), percentage differences between $TWMC_{PS}$ and $TWMC_{HFS}$ between $TWMC_{PS}$ and $FWMC_{HFS}$ were assessed against changing river discharge conditions (Jordan et al. 2013). Multiple regressions (using the “lm” function in the R “stats” package), using percentage differences as the dependent variable, included river discharge metrics (Q_{min}, Q_{max}, Q_{median}, and Q_{mean}) on data from both sites separately and combined. Variance inflation factors (VIFs) were used to assess collinearity between predictor variables (using the “vif” function in the “car” package (Fox and Weisbert 2019)) and the variables with the highest VIFs were removed stepwise until all VIFs were below 1/(1-R^2 of model) or until only two variables were left (single regressions were also checked). Residuals versus fitted values for the models were examined graphically for normality.

References

Fox, J. and Weisbert, S. (2019) An R Companion to Applied Regression, Sage, Thousand Oaks CA.

Hothorn, T., Bretz, F., Westfall, P., Heiberger, R.M., Schuetzenmeister, A. and Scheibe, S. (2008) Simultaneous Inference in General Parametric Models. Biometrical Journal 50(3), 346 - 363.

Jordan, P., Cassidy, R., Macintosh, K.A. and Arnscheidt, J. (2013) Field and Laboratory Tests of Flow-Proportional Passive Samplers for Determining Average Phosphorus and Nitrogen Concentration in Rivers. Environmental Science & Technology 47(5), 2331-2338.

Kuznetsova, A., Brockhoff, P. and Christensen, R. (2017) lmerTest Package: Tests in Linear Mixed Effects Models. Journal of Statistical Software 82, 1-26.

R Development Core Team (2020) R: A language and environment for statistical computing, Vienna, Austria.

Spilke, J., Piepho, H.-P. and Hu, X. (2005) A simulation study on tests of hypotheses and confidence intervals for fixed effects in mixed models for blocked experiments with missing data. Journal of Agricultural, Biological, and Environmental Statistics 10(3), 374-389.

Zuur, A.F., Leno, E.N., Walker, N., Saveliev, A.A. and Smith, G.M. (2009) Mixed effects models and extensions in ecology with R, Springer, New York, NY.
