# Supplementary material for: Evaluation of Chemcatcher® passive samplers for pesticide monitoring using high-frequency catchment scale data
Source: J Environ Manage. 2022 Dec 15;324:116292. doi: 10.1016/j.jenvman.2022.116292 (PMC9666346; doi:10.1016/j.jenvman.2022.116292)
Supplement: Multimedia component 2 [file mmc2.pdf]

Supplementary material

Table S.1: High frequency monitoring (HFS) sample collection frequency throughout the period of the study.

Start date of period	End date of period	One sample collected every…
30/10/18	11/12/18	7 hours
11/12/18	12/03/19	24 hours
12/03/19	10/12/19	7 hours
10/12/19	25/02/20	24 hours

Table S.2: The limits of detection for each of the acid herbicides under each monitoring technique

Herbicide	HFS limit of detection (ng L^{-1}) 30/10/18 – 25/02/20	Chemcatcher® limit of detection (ng L^{-1}) 30/10/18 – 09/07/19	Chemcatcher® limit of detection (ng L^{-1}) 23/07/19 – 20/02/20
MCPA	2.5	0.3	0.2
Triclopyr	1.0	0.3	0.2
Mecoprop	0.5	0.2	0.2
Fluroxypyr	0.5	0.5	0.4

Table S.3 Dates between which Chemcatchers were deployed

Start date of period	End date of period
30/10/18	11/12/18
08/01/19	09/07/19
23/07/19	10/12/19
14/01/20	20/02/20
