# Supplementary material for: Evaluation of Chemcatcher® passive samplers for pesticide monitoring using high-frequency catchment scale data
Source: J Environ Manage. 2022 Dec 15;324:116292. doi: 10.1016/j.jenvman.2022.116292 (PMC9666346; doi:10.1016/j.jenvman.2022.116292)
Supplement: Multimedia component 3 [file mmc3.pdf]

Period		ISCO				Chemcatcher		ISCO		Chemcatcher	
Start date	End date	FWMC (ng/L) - Derg	FWMC (ng/L) - Finn	TWMC (ng/L) - Derg	TWMC (ng/L) - Finn	TWMC (ng/L) - Derg	TWMC (ng/L) - Finn	TotalLoad (kg) - Derg	TotalLoad (kg) - Finn	TotalLoad (kg) - Derg	TotalLoad (kg) - Finn
30/10/2018	13/11/2018	67.04	19.62	52.27	16.24	43.15		1.32	0.51	0.88	
13/11/2018	27/11/2018	33.17	16.25	13.55	17.67	11.75	14.20	0.55	0.26	0.20	0.23
27/11/2018	11/12/2018	33.20	32.73	27.41	29.01	23.22	49.80	1.24	1.36	0.84	2.07
11/12/2018	25/12/2018	10.36	7.05	9.76	6.41			0.30	0.22		
25/12/2018	08/01/2019	4.52	5.13	5.20	5.32			0.05	0.06		
08/01/2019	22/01/2019	4.92	10.13	6.03	11.49	2.95	11.20	0.06	0.17	0.04	0.19
22/01/2019	05/02/2019	7.02	7.70	7.77	9.53	6.63	10.50	0.17	0.22	0.16	0.30
05/02/2019	19/02/2019	14.13	3.67	12.00	8.53	13.50	8.80	0.47	0.16	0.41	0.33
19/02/2019	05/03/2019	6.30	10.87	8.19	11.47	8.34	31.50	0.09	0.15	0.01	0.45
05/03/2019	19/03/2019	24.97	34.97	24.67	36.52	19.78	50.50	1.61	2.19	1.42	3.16
19/03/2019	02/04/2019	6.44	8.86	10.73	7.52	5.88	7.60	0.08	0.11	0.07	0.09
02/04/2019	16/04/2019	86.85	16.67	36.13	10.96	34.07	12.10	0.76	0.20	0.29	0.15
16/04/2019	30/04/2019	385.03	331.68	148.76	141.91	137.37	149.40	1.46	1.83	0.52	0.83
30/04/2019	14/05/2019	649.06	73.81	463.14	76.84	348.54	80.90	2.93	0.37	1.56	0.41
14/05/2019	28/05/2019	90.29	78.43	60.95	75.87	78.36	93.80	0.18	0.25	0.16	0.30
28/05/2019	11/06/2019	734.07	573.00	493.45	419.20	618.81	554.30	23.99	18.22	20.32	17.62
11/06/2019	25/06/2019	273.06	705.34	212.38	502.64	185.95	668.40	1.29	5.96	0.87	5.65
25/06/2019	09/07/2019	66.50	96.95	69.52	98.23	66.89	104.50	0.12	0.34	0.12	0.36
09/07/2019	23/07/2019	450.79	345.76	292.66	306.89			8.17	5.64		
23/07/2019	06/08/2019	680.98	305.67	243.64	287.63	255.24	286.08	4.97	1.97	1.89	1.85
06/08/2019	20/08/2019	579.55	474.32	381.58	471.56	306.84	531.40	11.96	15.50	6.20	17.36
20/08/2019	03/09/2019	374.12	293.71	268.40	237.35	260.60	146.20	15.65	15.54	10.88	6.97
03/09/2019	17/09/2019	77.29	146.51	73.96	121.88	32.86	185.11	1.89	4.14	0.81	5.05
17/09/2019	01/10/2019	469.92	744.05	357.91	536.57	219.19	321.02	9.67	17.59	4.58	7.76
01/10/2019	15/10/2019	41.61	156.31	33.01	117.42	26.23	130.90	1.47	5.55	0.94	4.65
15/10/2019	29/10/2019	20.83	29.98	14.59	29.08	6.84	20.78	0.32	0.60	0.11	0.41
29/10/2019	12/11/2019	82.34	123.96	49.15	153.29	26.13	107.65	1.99	3.10	0.63	2.75
12/11/2019	26/11/2019	12.59	38.26	9.11	35.01	3.82	21.92	0.14	0.67	0.04	0.36
26/11/2019	10/12/2019	27.15	16.34	15.79	16.19	10.57	13.33	0.84	0.56	0.33	0.46
10/12/2019	24/12/2019	7.92	10.78	8.36	12.42			0.29	0.48		
24/12/2019	07/01/2020	5.18	8.83	5.37	9.09			0.09	0.16		
07/01/2020	14/01/2020										
14/01/2020	28/01/2020	3.56	10.48	4.43	11.71	1.76	4.43	0.05	0.22	0.03	0.09
28/01/2020	11/02/2020	7.21	13.00	6.71	13.58	2.98	7.96	0.30	0.65	0.12	0.38
11/02/2020	25/02/2020	5.10	13.65	6.03	13.58	1.68	8.18	0.36	1.02	0.11	0.58
