# Supplementary material for: Evaluation of Chemcatcher® passive samplers for pesticide monitoring using high-frequency catchment scale data
Source: J Environ Manage. 2022 Dec 15;324:116292. doi: 10.1016/j.jenvman.2022.116292 (PMC9666346; doi:10.1016/j.jenvman.2022.116292)
Supplement: Multimedia component 4 [file mmc4.pdf]

Period		ISCO				Chemcatcher		ISCO		Chemcatcher	
Start date	End date	FWMC (ng/L) - Derg	FWMC (ng/L) - Finn	TWMC (ng/L) - Derg	TWMC (ng/L) - Finn	TWMC (ng/L) - Derg	TWMC (ng/L) - Finn	TotalLoad (kg) - Derg	TotalLoad (kg) - Finn	TotalLoad (kg) - Derg	TotalLoad (kg) - Finn
30/10/2018	13/11/2018	12.00	1.25	5.83	1.28	4.16		0.24	0.03	0.08	
13/11/2018	27/11/2018	2.80	1.28	1.75	1.28	1.69	0.50	0.05	0.02	0.03	0.01
27/11/2018	11/12/2018	3.05	1.22	2.96	1.28	2.68	1.20	0.11	0.05	0.10	0.05
11/12/2018	25/12/2018	2.92	1.28	3.41	1.35			0.09	0.04		
25/12/2018	08/01/2019	1.79	1.20	2.00	1.35			0.02	0.01		
08/01/2019	22/01/2019	1.82	1.16	2.21	1.35	1.39	0.41	0.02	0.02	0.02	0.01
22/01/2019	05/02/2019	1.51	1.11	1.75	1.35	1.91	0.40	0.04	0.03	0.05	0.01
05/02/2019	19/02/2019	3.04	0.61	2.44	1.67	3.42	0.80	0.10	0.03	0.10	0.03
19/02/2019	05/03/2019	1.59	0.86	2.30	1.35	2.23	0.50	0.02	0.01	0.04	0.01
05/03/2019	19/03/2019	4.83	1.17	4.48	1.28	3.18	0.70	0.31	0.07	0.23	0.04
19/03/2019	02/04/2019	1.33	1.24	1.46	1.28	1.10	0.30	0.02	0.02	0.01	0.00
02/04/2019	16/04/2019	5.14	1.71	4.11	2.34	2.58	0.60	0.04	0.02	0.02	0.01
16/04/2019	30/04/2019	30.14	51.79	32.61	80.84	14.71	6.80	0.11	0.29	0.06	0.04
30/04/2019	14/05/2019	47.35	12.46	31.83	13.02	17.57	4.50	0.21	0.06	0.08	0.02
14/05/2019	28/05/2019	20.67	7.10	19.69	8.01	14.26	3.40	0.04	0.02	0.03	0.01
28/05/2019	11/06/2019	32.08	10.26	30.91	9.03	25.06	6.20	1.05	0.33	0.82	0.20
11/06/2019	25/06/2019	31.74	13.82	32.17	11.68	15.33	6.00	0.15	0.12	0.07	0.05
25/06/2019	09/07/2019	19.72	21.69	20.95	23.84	7.37	2.60	0.03	0.08	0.01	0.01
09/07/2019	23/07/2019	92.96	10.58	65.25	11.14			1.68	0.17		
23/07/2019	06/08/2019	47.89	16.08	29.82	16.61	13.85	6.74	0.35	0.10	0.10	0.04
06/08/2019	20/08/2019	36.46	14.03	27.30	11.33	11.32	6.06	0.75	0.46	0.23	0.20
20/08/2019	03/09/2019	33.70	9.33	19.44	10.22	7.75	4.97	1.41	0.49	0.32	0.24
03/09/2019	17/09/2019	16.12	15.79	15.97	11.78	1.42	2.20	0.39	0.45	0.04	0.06
17/09/2019	01/10/2019	28.95	37.30	29.61	31.14	5.33	6.32	0.60	0.88	0.11	0.15
01/10/2019	15/10/2019	16.51	10.62	15.60	7.81	2.90	2.09	0.58	0.38	0.10	0.07
15/10/2019	29/10/2019	10.91	5.71	11.31	5.60	2.43	0.61	0.17	0.11	0.04	0.01
29/10/2019	12/11/2019	10.54	4.34	9.40	4.91	1.94	1.13	0.25	0.11	0.05	0.03
12/11/2019	26/11/2019	10.71	8.72	11.81	8.70	1.45	0.54	0.12	0.15	0.02	0.01
26/11/2019	10/12/2019	6.12	3.75	6.09	3.57	0.99	0.68	0.19	0.13	0.03	0.02
10/12/2019	24/12/2019	12.75	13.40	14.72	15.34			0.46	0.60		
24/12/2019	07/01/2020	22.42	25.17	27.21	29.88			0.37	0.47		
07/01/2020	14/01/2020										
14/01/2020	28/01/2020	9.70	9.21	11.32	9.88	1.03	0.26	0.14	0.19	0.02	0.01
28/01/2020	11/02/2020	9.00	5.63	10.15	7.68	1.09	0.59	0.37	0.28	0.04	0.03
11/02/2020	25/02/2020	10.46	10.83	12.65	12.69	1.00	0.60	0.73	0.81	0.07	0.04
