# Supplementary material for: Evaluation of Chemcatcher® passive samplers for pesticide monitoring using high-frequency catchment scale data
Source: J Environ Manage. 2022 Dec 15;324:116292. doi: 10.1016/j.jenvman.2022.116292 (PMC9666346; doi:10.1016/j.jenvman.2022.116292)
Supplement: Multimedia component 5 [file mmc5.pdf]

Period		ISCO				Chemcatcher		ISCO		Chemcatcher	
Start date	End date	FWMC (ng/L) - Derg	FWMC (ng/L) - Finn	TWMC (ng/L) - Derg	TWMC (ng/L) - Finn	TWMC (ng/L) - Derg	TWMC (ng/L) - Finn	TotalLoad (kg) - Derg	TotalLoad (kg) - Finn	TotalLoad (kg) - Derg	TotalLoad (kg) - Finn
30/10/2018	13/11/2018	147.86	1.77	136.45	1.68	53.60		2.92	0.05	1.09	
13/11/2018	27/11/2018	44.75	1.38	22.87	1.77	10.90	0.32	0.74	0.02	0.19	0.01
27/11/2018	11/12/2018	31.02	1.57	27.10	1.66	13.50	0.37	1.16	0.07	0.49	0.02
11/12/2018	25/12/2018	4.47	1.17	4.74	1.42			0.13	0.04		
25/12/2018	08/01/2019	2.30	1.88	2.68	2.18			0.02	0.02		
08/01/2019	22/01/2019	1.63	0.96	2.11	1.11	0.44	0.10	0.02	0.02	0.01	0.00
22/01/2019	05/02/2019	1.55	1.47	1.88	1.82	0.50	0.29	0.04	0.04	0.01	0.01
05/02/2019	19/02/2019	2.57	0.76	2.48	1.98	0.63	0.43	0.08	0.03	0.02	0.02
19/02/2019	05/03/2019	1.28	0.92	2.06	1.37	0.43	0.32	0.02	0.01	0.01	0.00
05/03/2019	19/03/2019	2.67	0.89	2.62	0.96	0.82	0.35	0.17	0.06	0.06	0.02
19/03/2019	02/04/2019	1.36	0.65	1.42	0.79	0.36	0.31	0.02	0.01	0.00	0.00
02/04/2019	16/04/2019	3.10	5.46	2.34	3.11	0.72	1.76	0.03	0.07	0.01	0.02
16/04/2019	30/04/2019	15.73	2.25	6.56	1.49	2.41	0.81	0.06	0.01	0.01	0.00
30/04/2019	14/05/2019	32.75	0.73	19.37	0.74	5.67	0.31	0.15	0.00	0.03	0.00
14/05/2019	28/05/2019	4.90	1.01	5.57	1.02	2.37	0.40	0.01	0.00	0.00	0.00
28/05/2019	11/06/2019	11.79	1.95	10.77	1.62	5.09	0.64	0.39	0.06	0.17	0.02
11/06/2019	25/06/2019	2.07	1.89	2.01	1.72	0.75	0.77	0.01	0.02	0.00	0.01
25/06/2019	09/07/2019	2.39	1.81	2.52	1.84	0.57	0.50	0.00	0.01	0.00	0.00
09/07/2019	23/07/2019	4.10	1.83	2.90	1.35			0.07	0.03		
23/07/2019	06/08/2019	15.23	2.07	6.53	1.92	6.02	0.93	0.11	0.01	0.04	0.01
06/08/2019	20/08/2019	10.17	1.29	7.27	1.20	5.71	0.60	0.21	0.04	0.12	0.02
20/08/2019	03/09/2019	3.17	0.73	2.87	0.90	2.45	1.13	0.13	0.04	0.10	0.05
03/09/2019	17/09/2019	2.14	1.85	2.25	1.75	0.91	0.52	0.05	0.05	0.02	0.01
17/09/2019	01/10/2019	20.59	2.14	15.34	1.69	7.09	0.64	0.42	0.05	0.15	0.02
01/10/2019	15/10/2019	4.11	1.94	3.71	1.73	2.38	0.97	0.14	0.07	0.09	0.03
15/10/2019	29/10/2019	2.44	1.11	2.11	1.07	0.89	0.26	0.04	0.02	0.01	0.01
29/10/2019	12/11/2019	10.18	1.27	6.08	1.00	2.89	0.93	0.25	0.03	0.07	0.02
12/11/2019	26/11/2019	1.85	0.79	1.80	0.98	0.72	0.30	0.02	0.01	0.01	0.00
26/11/2019	10/12/2019	1.66	0.63	1.82	0.62	0.60	0.38	0.05	0.02	0.02	0.01
10/12/2019	24/12/2019	3.03	0.46	3.42	0.54			0.11	0.02		
24/12/2019	07/01/2020	2.53	0.74	2.73	0.69			0.04	0.01		
07/01/2020	14/01/2020										
14/01/2020	28/01/2020	2.60	3.33	3.23	3.54	0.75	0.97	0.04	0.07	0.01	0.02
28/01/2020	11/02/2020	3.34	3.27	4.22	4.23	0.66	0.52	0.14	0.16	0.03	0.02
11/02/2020	25/02/2020	3.11	2.11	3.65	2.19	0.73	0.37	0.22	0.16	0.05	0.03
