# Supplementary material for: Evaluation of Chemcatcher® passive samplers for pesticide monitoring using high-frequency catchment scale data
Source: J Environ Manage. 2022 Dec 15;324:116292. doi: 10.1016/j.jenvman.2022.116292 (PMC9666346; doi:10.1016/j.jenvman.2022.116292)
Supplement: Multimedia component 6 [file mmc6.pdf]

Period		ISCO				Chemcatcher		ISCO		Chemcatcher	
Start date	End date	FWMC (ng/L) - Derg	FWMC (ng/L) - Finn	TWMC (ng/L) - Derg	TWMC (ng/L) - Finn	TWMC (ng/L) - Derg	TWMC (ng/L) - Finn	TotalLoad (kg) - Derg	TotalLoad (kg) - Finn	TotalLoad (kg) - Derg	TotalLoad (kg) - Finn
30/10/2018	13/11/2018	4.78	0.69	2.84	0.63	2.00		0.09	0.02	0.04	
13/11/2018	27/11/2018	1.35	0.98	1.07	0.80	0.70	0.60	0.02	0.02	0.01	0.01
27/11/2018	11/12/2018	1.57	0.49	1.54	0.51	1.50	1.30	0.06	0.02	0.05	0.05
11/12/2018	25/12/2018	1.38	0.95	1.72	1.02			0.04	0.03		
25/12/2018	08/01/2019	0.88	0.70	0.94	0.75			0.01	0.01		
08/01/2019	22/01/2019	1.27	0.50	1.61	0.59	0.90	0.50	0.02	0.01	0.01	0.01
22/01/2019	05/02/2019	1.35	0.60	1.32	0.63	1.10	0.60	0.03	0.02	0.03	0.02
05/02/2019	19/02/2019	1.57	0.59	1.61	1.57	1.20	1.00	0.05	0.03	0.04	0.04
19/02/2019	05/03/2019	0.91	0.44	1.20	0.66	1.10	0.60	0.01	0.01	0.02	0.01
05/03/2019	19/03/2019	3.66	0.85	3.33	0.99	1.90	0.80	0.24	0.05	0.14	0.05
19/03/2019	02/04/2019	0.94	0.49	1.09	0.51	0.25	0.25	0.01	0.01	0.00	0.00
02/04/2019	16/04/2019	3.57	1.42	2.57	1.18	1.50	0.70	0.03	0.02	0.01	0.01
16/04/2019	30/04/2019	14.15	5.65	13.20	7.31	8.30	4.40	0.05	0.03	0.03	0.02
30/04/2019	14/05/2019	28.09	3.49	18.74	3.73	8.30	1.40	0.13	0.02	0.04	0.01
14/05/2019	28/05/2019	12.84	2.97	10.44	3.48	6.40	0.80	0.03	0.01	0.01	0.00
28/05/2019	11/06/2019	19.94	5.01	17.45	4.15	12.50	1.60	0.65	0.16	0.41	0.05
11/06/2019	25/06/2019	15.06	8.37	15.08	7.21	7.40	2.50	0.07	0.07	0.03	0.02
25/06/2019	09/07/2019	10.85	10.44	11.27	11.45	4.60	0.70	0.02	0.04	0.01	0.00
09/07/2019	23/07/2019	28.65	5.04	27.67	6.85			0.52	0.08		
23/07/2019	06/08/2019	26.18	7.21	15.72	7.79	8.97	3.41	0.19	0.05	0.07	0.02
06/08/2019	20/08/2019	21.77	6.21	16.66	5.34	8.02	2.21	0.45	0.20	0.16	0.07
20/08/2019	03/09/2019	14.34	3.91	9.20	3.79	6.05	1.75	0.60	0.21	0.25	0.08
03/09/2019	17/09/2019	6.17	6.88	5.81	5.29	0.99	0.93	0.15	0.19	0.02	0.03
17/09/2019	01/10/2019	18.97	14.65	18.69	12.82	4.44	2.16	0.39	0.35	0.09	0.05
01/10/2019	15/10/2019	8.69	4.68	8.74	3.66	2.19	1.02	0.31	0.17	0.08	0.04
15/10/2019	29/10/2019	5.73	2.97	5.16	2.66	1.28	0.42	0.09	0.06	0.02	0.01
29/10/2019	12/11/2019	6.18	2.20	4.90	2.64	1.36	0.81	0.15	0.05	0.03	0.02
12/11/2019	26/11/2019	4.24	4.04	4.20	3.73	0.65	0.20	0.05	0.07	0.01	0.00
26/11/2019	10/12/2019	3.52	2.46	3.72	2.06	0.79	0.77	0.11	0.08	0.02	0.03
10/12/2019	24/12/2019	3.69	10.47	4.32	11.43			0.13	0.47		
24/12/2019	07/01/2020	10.31	14.44	13.00	16.03			0.17	0.27		
07/01/2020	14/01/2020										
14/01/2020	28/01/2020	9.08	6.03	10.46	6.58	1.00	0.20	0.13	0.13	0.02	0.00
28/01/2020	11/02/2020	4.49	2.52	5.24	2.99	1.04	0.97	0.19	0.13	0.04	0.05
11/02/2020	25/02/2020	8.69	9.01	10.27	10.73	0.90	0.79	0.61	0.68	0.06	0.06
